# Roles of Germin-like Protein Family in Response to Seed Germination and Shoot Branching in *Brassica napus*

**DOI:** 10.3390/ijms252111518

**Published:** 2024-10-26

**Authors:** Qian Zhang, Luman Wang, Xinfa Wang, Jiangwei Qiao, Hanzhong Wang

**Affiliations:** Key Laboratory of Biology and Genetic Improvement of Oil Crops, Oil Crops Research Institute of the Chinese Academy of Agricultural Sciences, Ministry of Agriculture and Rural Affairs, Wuhan 430062, China

**Keywords:** *Brassica napus*, *GLPs* family, seed germination, shoot branching

## Abstract

Germin-like proteins (GLPs) play important roles in the regulation of various plant development processes, such as seed vigor, root and leaf development and disease resistance, while the roles of *GLPs* on agronomic traits are rarely studied in *Brassica napus*. Here, we identified *GLPs* family genes in rapeseed and analyzed their potential functions. There are 77 *GLPs* family genes (*BnGLPs*) in the Zhongshuang11 rapeseed reference genome, divided into a, b, c, d, e, f six subfamilies. Tissue expression profile analysis of *BnGLPs* revealed the following: e subfamily genes were highly expressed in early stages of silique, cotyledon, vegetative rosette and leaf development; f subfamily genes were highly expressed in seed development; genes of a subfamily were mainly expressed in the root; and genes of b, c, d subfamily exhibited low-level or no expression in above mentioned tissues. RT-qPCR analysis confirmed that the transcripts of two f subfamily members decreased dramatically during seed germination, suggesting that f subfamily proteins may play vital roles in the early stage of seed germination. Transcriptome analysis of axillary buds in sequential developing stages revealed that the transcripts of eight e subfamily genes showed a rapid increase at the beginning of shoot branching, implying that the e subfamily members played vital roles in branch development. These results demonstrate that rapeseed *BnGLPs* likely play essential roles in seedling development, root development and plant architecture, indicating that harnessing certain *BnGLPs* may contribute to the improvement of rapeseed yield.

## 1. Introduction

Cupin proteins were discovered through an unusually thermostable protein produced early in the germination of wheat embryos and named “germin” because of its function [1]. Germin-like proteins (GLPs) containing a single cupin_1 domain which belongs to monocupin proteins are markers of plant germination [1,2,3]. GLPs are homohexameric glycoproteins that contain lengths of 140-150 amino acids conserved cupin_1 domain with metal ion binding at the C-terminus [3]. *GLPs* have various functions at different developmental stages and have been reported to be associated with diverse functions, such as plant development and plant defense [4,5,6]. The exploration of the entire process of plum (*Prunus salicina*) flower and fruit development has shown that *PsGLP1* and *PsGLP2* play important roles in these stages [7]. *GLPs* had been shown to be involved in plant pathogen resistance. *GmGLP10* positively regulated its defensive against *Sclerotinia Sclerotiorum* in transgenic tobacco, and *HaGLP1* had similar roles in *Arabidopsis* [8,9]. Furthermore, *OsGLP3-7* participated in plant pathogen resistance by affecting jasmonic acid (JA) and phytoalexin metabolic pathways [10].

*GLPs* family members are widely expressed in diverse plant organs (root, stem, leaf and flower), which have regulatory effects on different organs [11]. For example, PDGLP1 and PDGLP2 of *Arabidopsis* control phloem-mediated resource allocation between primary root and lateral root meristems to affect primary root growth [5]. In *Nicotiana tabacum*, *NtGLP1* is mainly expressed in leaves, especially in the elongation period, which may be related to the control of leaf cell space [12]. The research on rice has shown that *OsGLP2–1* plays an important role in seed dormancy, the expression of which was regulated by several key transcription factors of ABA and GA signaling pathways, thereby maintaining the seed primary dormancy process [4]. The seed germination capacity of rapeseed directly determines the morphogenesis of the seedlings and the later yield, which attracts considerable researcher’s interests. To clarify the roles of *BnGLPs* in *Brassica napus* (*B. napus*) seed germination, the gene expression patterns and the molecular mechanism of *BnGLPs* remain to be explored.

Branch derived from the growth of axillary buds, and are an important component determining the rapeseed yield [13]. However, the research on functional genes related to branching traits is relatively tardy, so there is still much research to be carried out in the study of rapeseed branching. Branch development initiates from the axillary meristems (AMS) of leaf axils, the AMS subsequently form into axillary buds, which then either remain dormant or continue to grow into branches [13,14]. The formation and development of axillary buds are systematically regulated by plant hormones, environmental factors and genetic factors [15,16]. GLPs are ubiquitous water-soluble glycoproteins which contain conserved N-glycosylation sites (NXS/T) associated with the JA-dependent pathway [11,17]. JA makes great differences in regulating branching, and negatively regulates branch growth in pear [18]. However, the relationship between GLPs and branching needs to be explored. In this study, we explored the potential relationship among GLPs, JA and branch development, by systematically analyzing the corresponding transcriptome data of axillary buds and the public gene-expression database of hormone treatment.

Rapeseed (*B. napus* L., AACC, 2n = 38) is one of the most important sources of edible oil for human consumption and oilcake for breeding [19,20]. At present, the whole genomic sequence of “Darmor-bzh” and “Zhongshuang11” (ZS11), which are representatives of two rapeseed ecotypes, have been finely determined. The cupin_1 domain containing proteins gene family had been reported in Darmor-bzh [19,21], to dissect the *GLPs* functions of shoot branching and seed germination in a semi-winter cultivator (e.g., ZS11) is meaningful. In this study, members of the *BnGLPs* family containing a single cupin-1 domain were systematically analyzed and a series of bioinformatic analyses were performed on these members. Based on the information consisting of tissue expression profiles, strong transcription of both during seed germination and in growing axillary buds, *BnGLPs* family members ought to play important roles in the rapeseed growth and development process. This study provides valuable insights for further investigation into the primary function of subfamily members, e subfamily members’ roles in branching and the roles of f subfamily in seed germination of rapeseed.

## 2. Results

### 2.1. Identification of BnGLPs Family Members in B. napus

**Figure 1:**
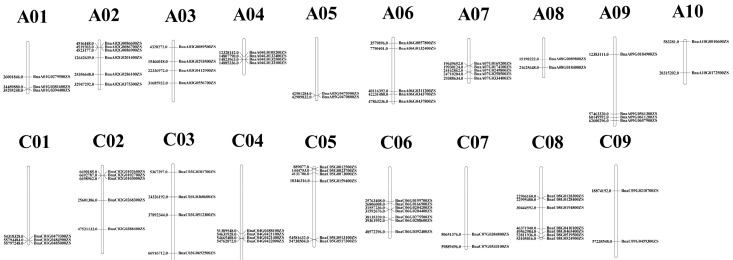
Distribution of *GLPs* on *B. napus* chromosomes. In total, 77 *BnGLPs* were mapped on 18 chromosomes.

The HMMER search and BLASTp analysis were used to search the genome of one Chinese semi-winter rapeseed cultivator (ZS11), 77 members of the *BnGLPs* were identified in *B. napus*, all of which contain the conserved structural domain cupin-1. Thirty-seven {*BnGLPs* were located in the A subgenome and the remaining 40 in the C subgenome (Table 1). Overall, *BnGPLs* distributed unevenly across ZS11 chromosomes, as shown in Figure 1. Specifically, there were 7, 7, 6 and 6 *BnGPLs* located on chromosomes C08, C06, A02 and C05, respectively. The length of BnCLPs varied from 88 amino acids (BnaC06T0204400ZS) to 703 amino acids (BnaA06T0311200ZS), with an average length of 242 amino acids. The predicted theoretical isoelectric point (pI) values varied from 4.77 to 9.64 and the molecular weight (MW) values were between 9636.31 and 84,184.33 Da. Moreover, the instability indexes of 11 members were found to be more than 40, indicating that they are likely to be unstable proteins. In particular one protein, BnaA06T0311200ZS, had a high instability index of 92.59. The grand average of hydrophobicity (Gravy) values ranged from –1.332 to 0.598, and GRAVY values of 63 members >0 predicted hydrophobic. According to the predicted subcellular location, the *BnGLPs* showed a wide distribution pattern across various subcellular locations. Specifically, they were found to be mainly located in the extracellular space (18/77) and chloroplasts (17/77) (Table 1).

### 2.2. Phylogenetic and Gene Duplication of Analysis BnGLPs

**Figure 2:**
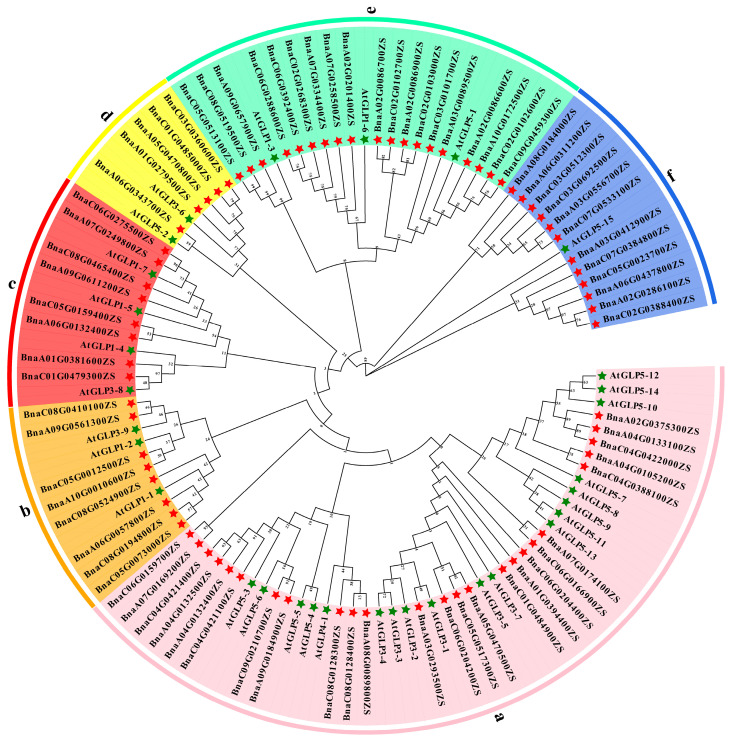
Phylogenetic analysis of Germ-like proteins from *B. napus* and *A. thaliana*. The amino acid sequences of 77 *BnGLPs* and 32 *AtGLPs* were aligned by the MUSCLE tool. A phylogenetic tree was generated by MEGA using the neighbor-joining (NJ) method (bootstrap replications, n = 1000). The phylogenetic tree was highlighted with Evolview (version 3.0). The proteins are clustered into six distinct clades which were designated clade a to f, respectively. These clades were labeled with different colors.

To further explore the characterizations and classifications of candidate *BnGLPs*, we constructed a phylogenetic tree which contains all 77 *BnGLPs* from the *B. napus* cultivator ZS11 and 32 *AtGLPs* from *Arabidopsis thaliana* (*A. thaliana*). All 77 *BnGLPs* varied significantly among the six subfamilies, with a subfamily contained the largest member of *BnGLPs* (25 genes), while subfamilies b–f only comprised 8, 8, 5, 19 and 12 *BnGLPs*, respectively (Figure 2). These results suggest that the *GLPs* of *Arabidopsis* and rapeseed had the common ancestors, some of which might have undergone species-specific expansion and significant divergence.

**Figure 3:**
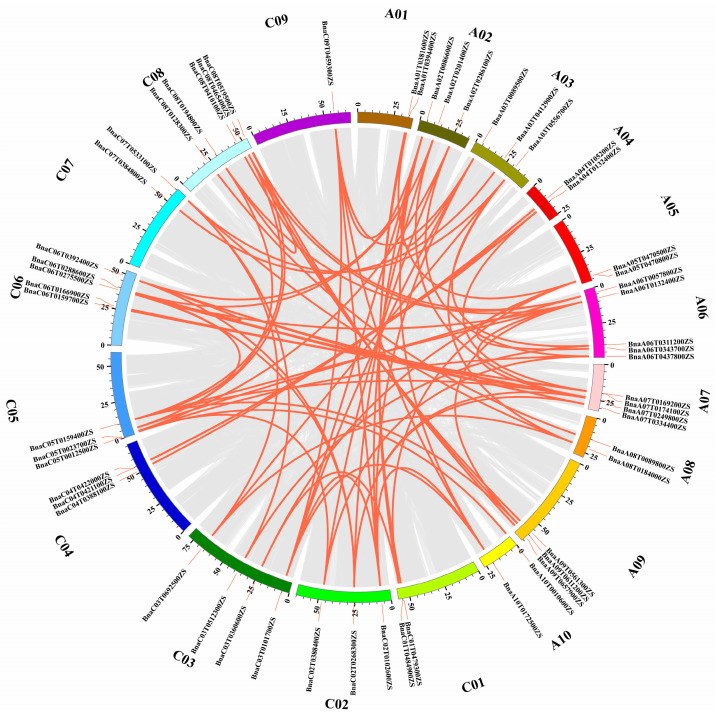
Genome-wide synteny analysis for *BnGLPs*. Gray lines indicate all the collinear blocks, and red lines highlight the orthologous relationships among *BnGLPs*.

Gene replication is crucial for the development of new genes and functions, and fragment and tandem repeats play significant roles in the expansion of gene families. The duplication events of *BnGLPs* were identified using BLAST and MCScan X. Specifically, out of the 77 *BnGLPs*, 80 pairs were found to be the result of segmental duplication events, while three pairs were tandem duplications (Figure 3 and Table S1). In total, there were 167 paralogous gene pairs with high identities (identity > 75% and alignment length > 75%) in *B. napus*. The ratio of non-synonymous substitution to synonymous substitution (Ka/Ks) was calculated for these 167 paralogous gene pairs. The results showed that the Ka/Ks ratio ranged from 0 to 0.83, all of which were lower than 1. This suggests that the *BnGLPs* have undergone purification selection during evolution (Table S2).

### 2.3. Gene Structure and Conserved Motif Analysis of the BnGLPs Family in B. napus

**Figure 4:**
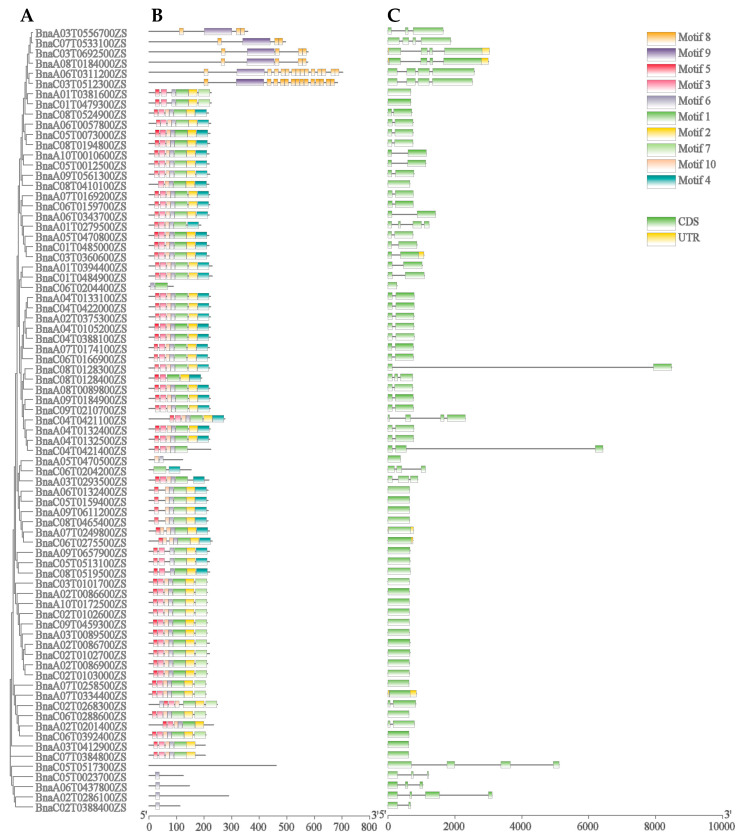
The phylogenetic relationship, exon-intron architecture and conserved motifs of 77 *BnGLPs* in *B. napus*. (**A**) The phylogenetic relationships of *BnGLPs* based on the NJ method. (**B**) The conserved motif composition of *BnGLPs*. (**C**) Gene structures of *BnGLPs*. Yellow boxes represent the untranslated regions (UTR), green boxes represent exons, and the gray lines represent introns.

To better comprehend the sequence diversity of the *BnGLPs* family in *B. napus*, we analyzed the gene structure and conserved motifs of *BnGLP*s. These *BnGLPs* have fewer numbers of introns and exons (61 *BnGLPs* contain one or two exons). By employing the MEME and TBtools software, 10 conserved protein motif types and locations were observed among 77 *BnGLPs*. Motif 1 and 5 existed on most *BnGLP*s, except the f subfamily which contained a maximum number of exons and introns (Figure 4 and Figure S1). Members of the same subfamily had nearly identical motifs, suggesting that they may be functionally identical or similar.

### 2.4. Spatial-Temporal Expressions of BnGLPs in Different Developmental Stages of B. napus 

**Figure 5:**
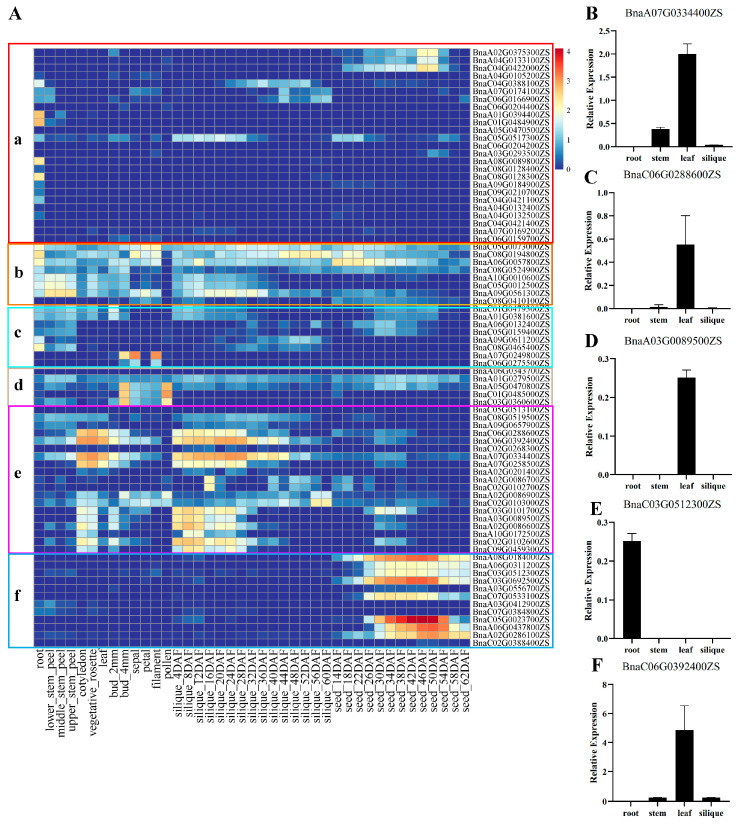
(**A**) Transcriptional expression profiles of 77 *BnGLPs* across different developmental stages and organs of ZS11 variety. (**B**–**F**) are relative expression levels of five *BnGLPs* in root, stem, leaf and silique. Error bars are standard deviations of three biological replicates. The color bar represents log10 expression values (Counts + 1). The color scale represents relative expression levels from low (blue color) to high (red color). DAF means days after flowering.

To understand the potential functions of *BnGLPs*, we analyzed the expression patterns of *BnGLPs* in 41 tissues including root, stem peel, cotyledon, leaf, bud, flower, silique and seed based on transcriptome data from BnIR [22]. And there exist 13 and 15 time points in seed and silique, respectively (Figure 5A). In total, 67 genes were expressed (fragments per kilobase million, FPKM > 1) in at least one developmental stage or one organ. Most genes in subfamily a, b, c and d were expressed at low levels in various tissues, except four genes (*BnaA01G0394400ZS*, *BnaC01G0484900ZS*, *BnaA08G0089800ZS*, *BnaC08G0128300ZS*) of a subfamily high expressing in root. Members in the e subfamily were highly expressed in cotyledon, vegetative rosette, leaf and early stages of silique development. Members in the f subfamily were highly expressed during the seed development process, particularly. The expression patterns of *BnGLPs* showed their involvements at the development stages of different organs in rapeseed. Genes within the same subfamily show similar expression patterns. The expression patterns of five genes in root, stem, leaf and silique of rapeseed (Figure 5B–F) are consistent with those in the heat map, supporting the reliability of *BnGLPs* expression patterns in the public database.

### 2.5. RT-qPCR Analysis of BnGLPs in Seed Germination

**Figure 6:**
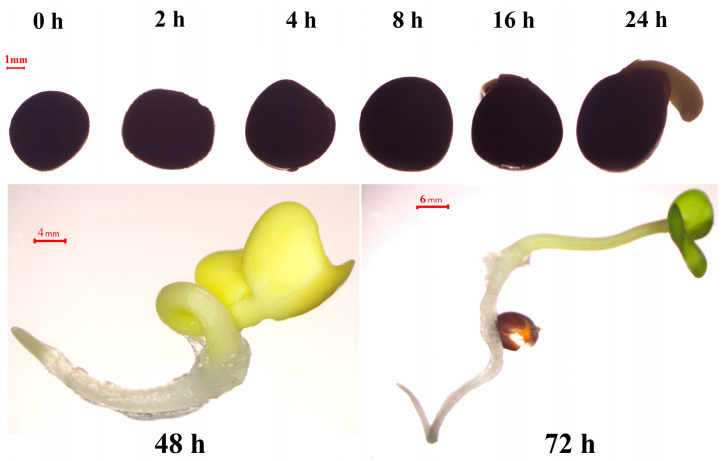
Seed germination of ZS11, where 0 h refers to dry seed; 2 h, 4 h, 8 h, 16 h, 24 h, 48 h and 72 h refer to the different stages of seed imbibed in water, respectively.

**Figure 7:**
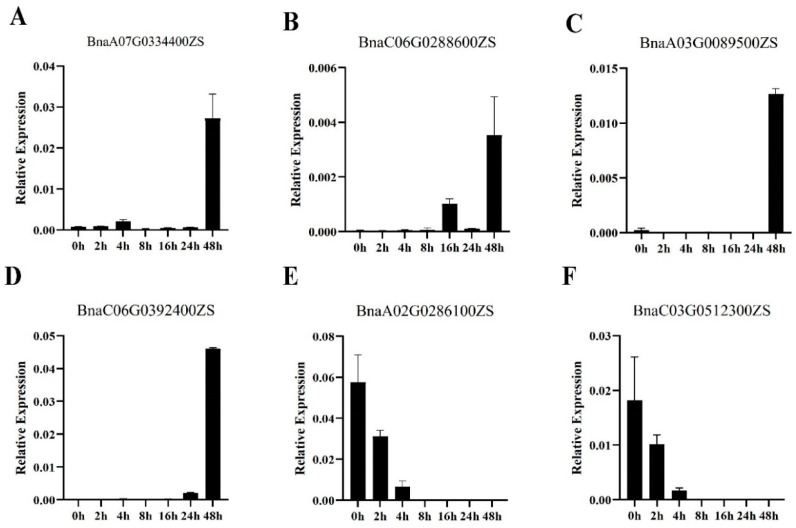
RT-qPCR analysis of expression differences in six genes at 0 h, 2 h, 4 h, 8 h, 16 h, 24 h and 48 h seed imbibed in water. Error bars are standard deviations of three biological replicates.

No germination was observed in 8 h water absorption of seeds, but the seeds already germinated after 16 h water absorption. In addition, after 24 h water absorption, all seeds have completely germinated (Figure 6 and Figure S2). Therefore, we chose the 0 h, 2 h, 4 h, 8 h, 16 h, 24 h and 48 h stages to explore four genes of the e subfamily and two genes of the f subfamily expression profiles in seed germination. The results showed that different subfamily genes have different expression profiles in seed germination (Figure 7). The relative expression level of e subfamily four members (*BnaA03G0089500ZS*, *BnaA07G0334400ZS*, *BnaC06G0288600ZS* and *BnaC06G0392400ZS*) were very seldom 0–24 h, but their relative expression level was pretty high in 48 h, which is the stage of seedling stage [23,24]. Nevertheless, two members (*BnaA02G0286100ZS* and *BnaC03G0512300ZS*) of the f subfamily are highly expressed in 0-4 h seed imbibed in water, especially in dry seeds (0 h).

**Figure 8:**
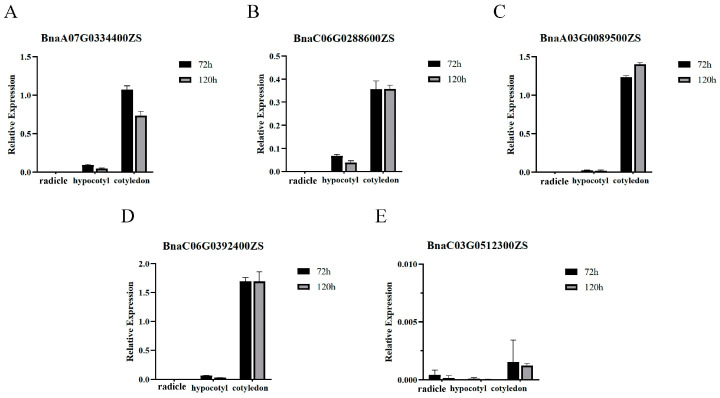
RT-qPCR analysis of five genes expression level expressed in radicles, hypocotyls and cotyledons after 72 h and 120 h seed imbibed in water. Error bars are standard deviations of three biological replicates.

The treated rapeseed seeds developed into seedlings after 48 h of water absorption and continued growing. We extracted RNA from radicles, hypocotyls and cotyledons after 72 h and 120 h seed imbibed in water for RT-qPCR analysis. The transcription levels of five genes at the seedling stage showed that four genes (*BnaA03G0089500ZS*, *BnaA07G0334400ZS*, *BnaC06G0288600ZS* and *BnaC06G0392400ZS*) of e subfamily highly expressed in cotyledons, and f subfamily gene *BnaC03G0512300ZS* barely expressed in rapeseed seedling (Figure 8). The expression of f subfamily gene *BnaA02G0286100ZS* was not detected for more than 45 cycles by fluorescence quantitative PCR. These results were consistent with the previous public transcriptome data.

### 2.6. Expression Patterns of BnGLPs in Four Different Development Stages of Axillary Buds

**Figure 9:**
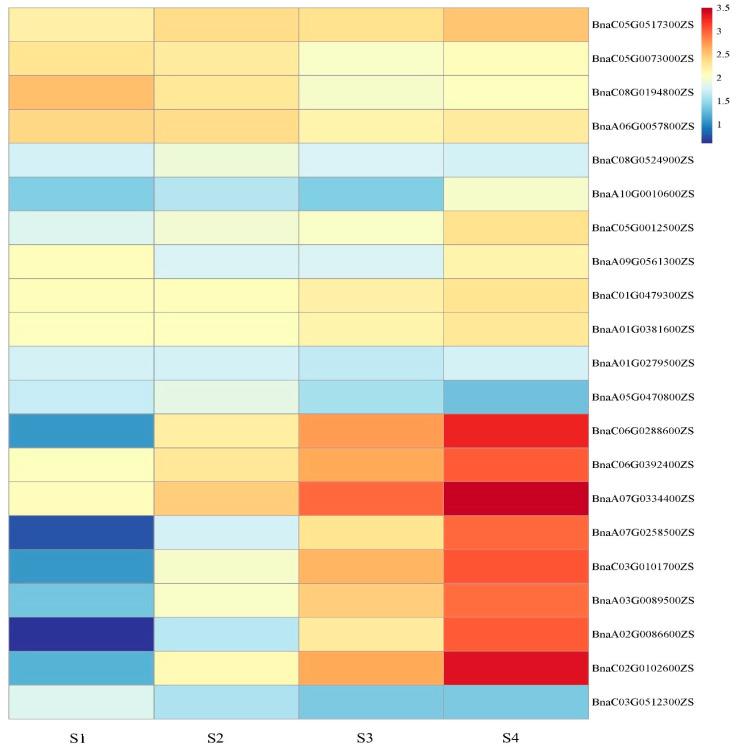
The expression of *BnGLPs* family members in different axillary buds. The color bar represents log10 expression values (Counts + 1) The color scale represents relative expression levels from low (blue color) to high (red color). S1 is the state of dormant axillary buds, S2 is the state of temporarily dormant axillary buds, S3 is the state of being activated axillary buds, S4 is the state of elongating axillary buds.

Shoot branching is a major factor affecting its architecture and yield [25]. To explore the potential roles of *GLPs* in shoot branching, axillary buds at four states (S1 to S4) were obtained from ZS11 plants and subjected to the RNA sequencing [26]. The *BnGLPs* with no expression or low expression level (FPKM < 1) were excluded for further analysis. We found that 21 *BnGLPs* were expressed during the process of axillary bud dormancy to activation. The result showed that the expression level of 8 *BnGLPs* (*BnaA02G0086600ZS*, *BnaA03G0089500ZS*, *BnaA07G0258500ZS*, *BnaA07G0334400ZS*, *BnaC02G0102600ZS*, *BnaC03G0101700ZS*, *BnaC06G0288600ZS* and *BnaC06G0392400ZS*) were the highest in elongating axillary buds (S4), activated axillary buds (S3), which belong to e subfamily (Figure 9). During the axillary buds elongation, the expression levels of these *BnGLPs* increased rapidly (Figure 9). These results indicate that members of the e subfamily may positively regulate the outgrowth of axillary buds. In addition, the expression patterns of 5 genes were validated by RT-qPCR (Figure S3), which indicates expression patterns of *BnGLPs* RNA-sequenced data are reliable.

### 2.7. Expression Patterns of BnGLPs Under Four Hormones Treatments

**Figure 10:**
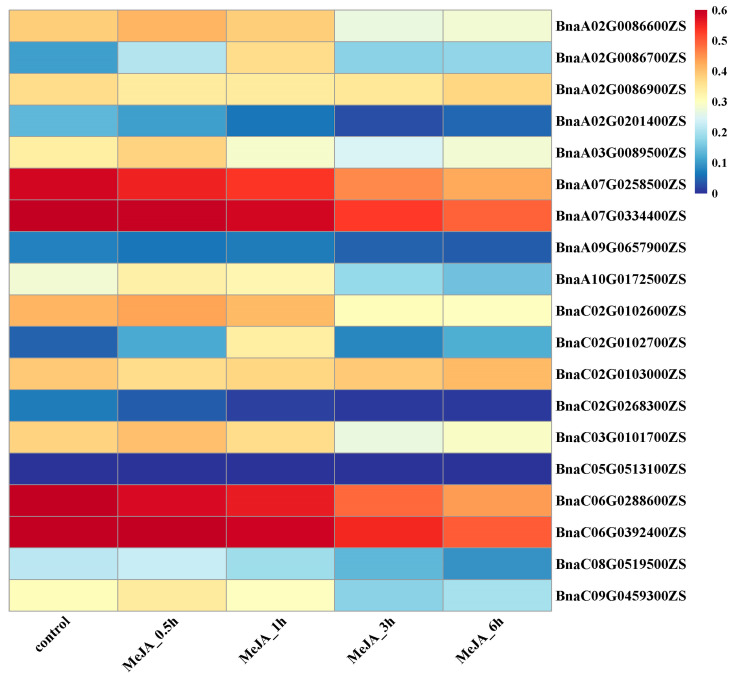
The expression of *BnGLPs* family members in leaf under MeJA hormone treatment. The color bar represents log10 expression values (Counts + 1). The color scale represents relative expression levels from low (blue color) to high (red color).

MeJA is a vital phytohormone and can greatly affect rapeseed seedling growth and plant development. We analyzed the expression of all e subfamily members in the leaf under MeJA hormone treatments, based on transcriptome data from BnIR [22]. The transcripts of 19 e subfamily genes, which maintain highly expressed in leaf (FPKM >1) (Figure 5), declined rapidly in leaf under JA hormone treatments (Figure 9). Eight of the 19 genes were found to be highly expressed in the activated and elongated axillary buds (Figure 10). Recently, JA was reported to negatively regulate branching in pear [18]. The results implied that e subfamily genes may be involved in JA-mediated negative regulation of branching in rapeseed.

## 3. Discussion 

*GLPs* are commonly found in plants and play crucial roles in plant growth, development and response to stress [27]. *GLPs* family has been identified in diverse plant species *A*. *thaliana*, rice, soybean, cucumber, potato, peanut and so on [10,28,29,30,31]. In this study, we conducted a comprehensive analysis of *BnGLPs* family in rapeseed. We examined various characteristics of *BnGLPs*, such as their phylogenetic classifications, chromosomal distributions, gene structures, conserved motifs, expression profiles and responses to hormone treatments. In this study, 77 *BnGLPs* were systematically identified in *B. napus* (ZS11 genome). A total of 3 pairs of tandem duplication genes and 80 pairs of segmental duplication genes were identified in the rapeseed *BnGLPs* family (Figure 3 and Table S1). Despite the uneven distribution of *BnCLPs* at the chromosome level, the total number of genes was roughly similar in A (37 members) and C subgenomes (40 members). Based on the Ka/Ks ratio of paralogous gene pairs (Table S2), it can be speculated that purification selection plays significant roles in the evolution of *BnGLPs* in rapeseed.

*GLP* family members have been confirmed to participate in the seed germination process [4]. Success in seed germination and seedling establishment determines the reproduction and survival of most plant species, and rapeseed seedling development is essential for profitable and sustainable production [32,33]. Seed germination includes three stages: (1) the imbibition stage, (2) the stage of increasing metabolic activity, (3) the stage of radicle breaking through the seed coat, which begins with water absorption and ends with the radicle breaking through the seed coat [23,34]. Our research showed that ZS11 seeds completed radicles breakthroughs in 24 h (Figure 6). Spatial-temporal expression results showed that only f subfamily members had specific and efficient transcriptional expression levels in seeds (Figure 5A). To explore the roles of members of the *BnGLPs* family in rapeseed seed germination, RT-qPCR analysis was carried out in the process of rapeseed germination. The results showed f subfamily members *BnaA02G0286100ZS* and *BnaC03G0512300ZS* expressed in early stage of seed germination (0–4 h seed imbibed in water), while the relative expression of e subfamily members *BnaA03G0089500ZS*, *BnaA07G0334400ZS*, *BnaC06G0288600ZS* and *BnaC06G0392400ZS* was highly expressed in seed imbibed in water with 48 h, at which the rapeseed seedling stage begins (Figure 7). Therefore, we speculated that f subfamily played key roles in seed germination. In addition, relative expression of these genes in radicles, hypocotyls and cotyledons of ZS11 seed imbibed in water with 72 h and 120 h showed that four e subfamily members (*BnaA03G0089500ZS*, *BnaA07G0334400ZS*, *BnaC06G0288600ZS* and *BnaC06G0392400ZS*) highly expressed in cotyledons, similar with the expression profile indicated by the rapeseed database BnIR [22] (Figure 8).

The *GLPs* affect seed germination and various plant development processes, thus may play important roles in crop improvement. Overexpression of a rice *GLPs* family member (*OsGER4*), which is related to the induction of crown root under exogenous JA treatment, increased the number of tillers and the number of grains per plant, and ultimately significantly increased the grain yield of rice [35]. In this study, transcriptional expression profiles of *BnGLPs* at different developmental stages and organs of ZS11 variety showed that four *BnGLPs* of a subfamily were highly expressed in root, which illustrates that *BnGLPs* may be involved in rapeseed root development. In addition, e subfamily *BnGLPs* are highly expressed in the early stages of silique, cotyledon, vegetative rosette and leaf development (Figure 4). Combined with the expression of *BnGLPs* by transcriptomic analysis of axillary buds in four states, we discovered that different subfamilies of *BnGLPs* may possess distinct roles in the growth and development of *B. napus* (Figure 10). Here, we found that e subfamily members may be involved in shoot branching. Transcriptomic analysis results showed the expression levels of eight e subfamily *BnGLPs* (*BnaA02G0086600ZS*, *BnaA03G0089500ZS*, *BnaA07G0258500ZS*, *BnaA07G0334400ZS*, *BnaC02G0102600ZS*, *BnaC03G0101700ZS*, *BnaC06G0288600ZS*, *BnaC06G0392400ZS*) at S4 stage of axillary buds is highest (Figure 10). What is more, the expression level of e subfamily members declined rapidly after 3 h JA treatments (Figure 9). Therefore, we supposed that e subfamily *BnGLPs* play important roles in axillary bud elongation during shooting branch.

In general conclusion, we inferred that rapeseed e subfamily and f subfamily *GLPs* play distinct roles in the growth and development of rapeseed, in which e subfamily *GLPs* involved in the regulation of leaf development and axillary bud elongation, while f subfamily *GLPs* play profound roles mainly in seed germination.

## 4. Materials and Methods

### 4.1. Plant Materials and Sampling

The semi-winter rapeseed cultivar “Zhongshuang11” (ZS11) was planted in Wuhan, China (113.68° E, 30.58° N). The intact seeds with same size were chosen and placed in a plate covered with two layers of filter paper, and sterile water was added to each plate, then cultivated in growth chamber at 22 °C, under 16/8 light-dark regime. Seeds of different seed germination states (0 h, 2 h, 4 h, 8 h, 16 h, 24 h, 48 h seed imbibed in water) were collected and cotyledons, hypocotyls, as well as radicles were collected after 72 h and 120 h seed imbibed in water. All of these samples were immediately frozen in liquid nitrogen and whereafter stored at −80 °C until RNA extraction.

### 4.2. RNA Extraction and RT-qPCR

Total RNA was extracted with Plant RNA Kit (R6827-01, Omega, GA, USA). cDNA was synthesized by PrimeScript RT reagent Kit with gDNA Eraser (RR047A, TaKaRa, Kyoto, Japan) and RT-qPCR was performed using a SYBR Premix Ex TaqTM II Kit (RR820A, TaKaRa, Kyoto, Japan) on a Roche LightCycler^®^ 96 instrument (Roche, Basel, Switzerland). The sequences of all RT-qPCR primers were shown in Table S3.

### 4.3. Identification and Phylogenetic Analyses of BnGLPs Gene Family in B. napus

A hidden Markov model (HMM) atlas of GLP (PF00190) was used to find predicted *GLPs* obtained from the BnTIR [22,36]. The protein sequences of 32 *AtGLPs* were utilized as queries and aligned with all protein sequences of rapeseed by running BLASTP on the website of BnTIR [22,36]. The Pfam database (http://pfam.xfam.org/, accessed on 7 July 2023), SMART database (http://smart.emblheidelberg.de, accessed on 7 July 2023) and NCBI conserved domain database (https://www.ncbi.nlm.nih.gov/cdd, accessed on 7 July 2023) were used to verify GLPs if there were complete cupin-1 domains [10]. The amino acids’ physicochemical properties of GLPs, such as the theoretical molecular weight (Mw), isoelectric point (pI), instability index and gravy, were predicted by ExPASy (https://www.expasy.org/, accessed on 10 September 2023). The phylogenetic tree of *AtGLPs* and *BnGLPs* was built by MEGA 7.0, which was based on alignments using the maximum likelihood method with 1000 boot-strap replicates [37]. The online editing software Evolview (https://www.evolgenius.info/evolview/#/treeview, accessed on 10 October 2024) was used to visualize the phylogenetic tree.

### 4.4. Gene Mapping and Synteny Analysis of BnGLPs in B. napus

Physical positions of *GLP* members on chromosomes from *B. napus* were drawn with the Mapchart [38]. The Multiple Collinearity Scan toolkit (MCScanX) was used to explore the collinearity relation among *BnGLPs* family members and visualized using Circos [39,40]. The KaKs Calculator program was used to calculate Ka/Ks of *BnGLPs* paralogous genes [41].

### 4.5. Gene Structure and Protein Motif Composition Analysis

The exon-intron structure of *GLPs* was determined by the Gene Structure Display Server (GSDS, http://gsds.cbi.pku.edu.cn/, accessed on 24 July 2023) algorithm with the genomic sequence of *BnGLPs* [42]. The amino acid sequence of *BnGLPs* was analyzed by the online program MEME (http://meme-suite.org/, accessed on 24 July 2023) to identify the conserved protein motifs [43]. The following parameters were employed in the analysis: the maximum number of motifs was 10, the minimum motif width was 6, and the maximum motif width was 100. The conserved motifs were further annotated with Pfam (http://pfam.xfam.org/, accessed on 24 July 2023).

### 4.6. Expression Profile Analysis Using Transcriptomic Data 

We explored the expression patterns of *BnGLPs* in rapeseed, based on public RNA-seq data from the *B. napus* multi-omics information resource (BnIR, https://yanglab.hzau.edu.cn/BnIR, accessed on 10 October 2024) [22]. This allowed us to analyze the biological functions of *BnGLPs*. The analysis of the expression of *BnGLPs* on different states of axillary buds (S1, S2, S3, S4) was based on our previous RNA-seq data [26]. The heat map depicting tissue-specific expression was generated using the OmicStudio tools at https://www.omicstudio.cn/tool, accessed on 3 September 2024 [40]. 

## 5. Conclusions

In this study, we identified and analyzed the *BnGLPs* family in the genome of one Chinese semi-winter rapeseed cultivator (ZS11). In total, 77 *BnGLPs* were identified and grouped into six subfamilies. Segmental duplication was found to be the major mode of family expansion during the evolution of *BnGLPs*. Spatial-temporal transcriptional expression analysis indicated that a subfamily *BnGLPs* may play roles in root development. The e subfamily *BnGLPs* may be involved in the growth and development of leaves and siliques. The f subfamily *BnGLPs* may be involved in seed development. The results of RT-qPCR analysis showed f subfamily *BnGLPs* highly expressed in the early stage of seed germination. Significantly, transcriptomic analysis of axillary buds and RT-qPCR analysis indicated e subfamily genes may likely play vital roles in elongation of axillary buds. These insights are applicable to further explore the distinct roles of *BnGLPs* genes in the rapeseed, the functional studies of which are meaningful and need to be conducted in future.

## Figures and Tables

**Table 1:** Features of the 77 *BnGLPs* identified in *B. napus*.

Gene ID	Chr	Start	End	Protein Length (aa)	MW (Da)	PI	Instability Index	Gravy	Subcellular Location
BnaA01G0279500ZS	A01	26001846	26003073	188	20,054.08	6.82	21.87	−0.015	Cytoplasm
BnaA01G0381600ZS	A01	34489880	34490560	226	24,720.46	6.7	27.42	−0.042	Chloroplast
BnaA01G0394400ZS	A01	35295249	35296270	229	24,842.54	6.41	25.47	0.239	Vacuole
BnaA02G0086600ZS	A02	4516448	4517083	211	21,894.19	6.39	37.63	0.328	Vacuole
BnaA02G0086700ZS	A02	4519203	4519862	219	22,632	5.76	35.83	0.4	Extracellular
BnaA02G0086900ZS	A02	4523177	4523815	212	22,087.47	7.81	38.16	0.411	Chloroplast
BnaA02G0201400ZS	A02	12642639	12643427	234	25,061.08	9.64	26.09	0.26	Chloroplast
BnaA02G0286100ZS	A02	25356649	25359760	289	32,052.41	6.59	55.66	−0.311	Extracellular
BnaA02G0375300ZS	A02	32947293	32948069	223	24,137.83	5.9	24.91	0.281	Peroxisome
BnaA03G0089500ZS	A03	4320272	4320907	211	21,843.12	6.4	33.52	0.387	Vacuole
BnaA03G0293500ZS	A03	15466017	15466904	217	23,281.77	9.27	29.76	0.164	Chloroplast
BnaA03G0412900ZS	A03	22336972	22337586	204	22,424.42	4.77	36.57	0.091	E.R.
BnaA03G0556700ZS	A03	31685932	31687583	358	40,189.81	5.92	58.48	−0.491	Cytoplasm
BnaA04G0105200ZS	A04	12328142	12328916	222	24,030.56	6.04	28.31	0.077	Extracellular
BnaA04G0132400ZS	A04	14807791	14808560	222	23,557.18	6.26	21.41	0.337	Chloroplast
BnaA04G0132500ZS	A04	14823963	14824726	220	23,365.93	6.26	20.08	0.323	Cytoplasm
BnaA04G0133100ZS	A04	14885336	14886115	223	24,079.75	5.9	25.59	0.307	Extracellular
BnaA05G0470500ZS	A05	42901285	42901653	122	13,547.99	6.81	22.82	−0.573	Chloroplast
BnaA05G0470800ZS	A05	42909832	42910578	217	23,067.51	7.8	23.95	−0.036	Extracellular
BnaA06G0057800ZS	A06	3579896	3580646	224	23,389.9	8.93	24.03	0.239	Peroxisome
BnaA06G0132400ZS	A06	7790401	7791045	214	22,469.75	5.85	28.96	0.32	Plasma membrane
BnaA06G0311200ZS	A06	40116394	40118973	703	84,184.33	5.7	92.59	−1.332	Chloroplast
BnaA06G0343700ZS	A06	42231478	42232896	218	23,173.89	9.42	32.01	0.226	Cytoplasm
BnaA06G0437800ZS	A06	47863237	47864278	147	16,243.68	9.22	48.09	−0.136	Vacuole
BnaA07G0169200ZS	A07	19649651	19650403	220	23,633.31	8.88	23.26	0.309	Extracellular
BnaA07G0174100ZS	A07	19930125	19930884	219	23,713.32	7.74	26.19	0.094	Cytoplasm
BnaA07G0249800ZS	A07	24162883	24163542	219	23,482.11	5.75	25.13	0.194	Chloroplast
BnaA07G0258500ZS	A07	24710205	24710828	207	21,620.23	9.23	26.48	0.521	Nuclearear
BnaA07G0334400ZS	A07	29388814	29389437	207	21,504.07	9.06	26.35	0.582	Extracellular
BnaA08G0089800ZS	A08	15198223	15198963	220	23,268.57	6.81	27.26	0.252	Chloroplast
BnaA08G0184000ZS	A08	21625853	21628613	576	64,442.47	5.47	54.41	−0.535	Plasma membrane
BnaA09G0184900ZS	A09	12383111	12383870	222	23,573.12	7.83	29.24	0.275	Plasma membrane
BnaA09G0561300ZS	A09	57463324	57464099	220	23,463.08	8.93	21.87	0.132	Cytoplasm
BnaA09G0611200ZS	A09	60149591	60150235	214	22,542.91	7.77	29.39	0.3	Cytoplasm
BnaA09G0657900ZS	A09	62600298	62600957	219	23,631.13	6.21	28.67	0.093	Extracellular
BnaA10G0010600ZS	A10	583281	584423	218	23,250.76	7.69	25.74	0.113	Cytoplasm
BnaA10G0172500ZS	A10	20315202	20315837	211	21,901.19	6.04	34.68	0.366	Peroxisome
BnaC01G0479300ZS	C01	54318329	54319009	226	24,595.34	6.7	26.38	−0.026	Vacuole
BnaC01G0484900ZS	C01	55794484	55795567	229	24,741.48	6.4	24.98	0.292	Chloroplast
BnaC01G0485000ZS	C01	55797246	55798113	218	23,192.56	6.5	24.73	0.047	Cytoplasm
BnaC02G0102600ZS	C02	6690185	6690820	211	21,895.17	6.04	38.62	0.328	Cytoplasm
BnaC02G0102700ZS	C02	6692787	6693446	219	22,597.98	5.76	33.85	0.402	Peroxisome
BnaC02G0103000ZS	C02	6698962	6699600	212	22,087.47	7.81	38.16	0.411	Extracellular
BnaC02G0268300ZS	C02	25601386	25602216	248	26,238.54	9.57	26.08	0.297	Extracellular
BnaC02G0388400ZS	C02	47521131	47521806	112	12,692.54	7.87	41.88	−0.047	Chloroplast
BnaC03G0101700ZS	C03	5367297	5367932	211	21,881.17	6.4	33.89	0.38	Extracellular
BnaC03G0360600ZS	C03	24326192	24327108	218	23,199.73	7.01	30.5	0.074	Extracellular
BnaC03G0512300ZS	C03	37892343	37894861	684	81,780.8	5.83	88.27	−1.271	Extracellular
BnaC03G0692500ZS	C03	66916733	66919518	577	64,785.96	5.49	58.48	−0.555	Chloroplast
BnaC04G0388100ZS	C04	51389547	51390329	222	24,000.48	6.04	27.95	0.066	Vacuole
BnaC04G0421100ZS	C04	54631928	54634237	275	29,706.55	8.43	19.74	0.265	Extracellular
BnaC04G0421400ZS	C04	54665409	54671832	224	24,000.68	7.74	15.65	0.109	Plasma membrane
BnaC04G0422000ZS	C04	54762870	54763648	223	24,096.77	5.64	24.85	0.331	Peroxisome
BnaC05G0012500ZS	C05	889077	890208	218	23,221.73	7.7	28.27	0.125	Chloroplast
BnaC05G0023700ZS	C05	1444753	1445964	124	13,871.84	5.62	45.81	0.002	Extracellular
BnaC05G0073000ZS	C05	4131706	4132454	221	23,244.79	9.17	26.25	0.206	Chloroplast
BnaC05G0159400ZS	C05	10346316	10346960	214	22,432.7	6.81	29.06	0.318	Chloroplast
BnaC05G0513100ZS	C05	54581632	54582291	219	23,713.18	6.42	30.34	0.013	Cytoplasm
BnaC05G0517300ZS	C05	54738504	54743617	461	50,045.61	9.42	45	0.103	Extracellular
BnaC06G0159700ZS	C06	25763408	25764160	220	23,674.3	8.64	25.89	0.267	Extracellular
BnaC06G0166900ZS	C06	26806007	26806766	219	23,701.3	6.82	25.72	0.107	Vacuole
BnaC06G0204200ZS	C06	31557235	31558350	153	16,791.47	7.82	36.42	0.226	Cytoplasm
BnaC06G0204400ZS	C06	31592677	31592943	88	9636.31	9.39	22.62	0.231	Cytoplasm
BnaC06G0275500ZS	C06	38135322	38136011	229	24,712.64	7.74	19.59	0.25	Chloroplast
BnaC06G0288600ZS	C06	39361993	39362616	207	21,622.25	9.23	23.01	0.545	Vacuole
BnaC06G0392400ZS	C06	48572295	48572918	207	21,565.15	9.03	22.36	0.598	Vacuole
BnaC07G0384800ZS	C07	50651376	50651990	204	22,399.43	4.85	37.77	0.083	Plasma membrane
BnaC07G0533100ZS	C07	59889494	59891371	495	55,430.54	5.3	51.25	−0.25	Vacuole
BnaC08G0128300ZS	C08	22966159	22974637	220	23,268.57	6.81	25.26	0.251	Cytoplasm
BnaC08G0128400ZS	C08	22995479	22996212	191	20,407.36	6.81	31.45	0.241	Vacuole
BnaC08G0194800ZS	C08	30444591	30445333	220	23,306.84	8.88	27.83	0.22	E.R.
BnaC08G0410100ZS	C08	46371939	46372592	217	22,961.71	9.52	25.67	0.155	Cytoplasm
BnaC08G0465400ZS	C08	49562986	49563630	214	22,548.96	7.77	29.14	0.289	Chloroplast
BnaC08G0519500ZS	C08	52811937	52812599	220	23,801.4	6.27	29.39	0.126	Plasma membrane
BnaC08G0524900ZS	C08	53105014	53105726	216	22,737.02	7.8	30.63	0.135	Cytoplasm
BnaC09G0210700ZS	C09	18874192	18874951	222	23,610.23	7.83	31.36	0.28	Vacuole
BnaC09G0459300ZS	C09	57238548	57239183	211	21,901.19	6.04	34.68	0.366	Extracellular

## Data Availability

Data are contained within the article and its Supplementary Files.

## Supplementary Materials

The following supporting information can be downloaded at: https://www.mdpi.com/article/10.3390/ijms252111518/s1.
