# Supplementary material for: Roles of Germin-like Protein Family in Response to Seed Germination and Shoot Branching in Brassica napus
Source: Int J Mol Sci. 2024 Oct 26;25(21):11518. doi: 10.3390/ijms252111518 (PMC11546990; doi:10.3390/ijms252111518)
Supplement: Supplementary file 1 [file ijms-25-11518-s001.zip › ijms-3214814-supplementary.pdf]

Roles of Germin-Like Protein Family in Response to Seed Germination and Shoot Branching in *Brassica napus*

Qian Zhang, Luman Wang, Xinfa Wang, Jiangwei Qiao * and Hanzhong Wang

The following Supporting Information is available for this article:

Supporting Figures:

Figure S1 The 10 conserved motifs of rapeseed *BnGLPs*.

Figure S2 0-4 h seed germination of ZS11.

Figure S3 Relative expression levels of 5 *BnGLPs* in different state of axillary buds.

Supporting Tables:

Table S1 Segmental duplication and tandem duplication gene pairs among *BnGLPs* family members.

Table S2 Ka/Ks analysis for paralogous gene pairs of *BnGLPs*.

Table S3 ZS11 *BnGLPs* gene-specific primers used for RT-qPCR analysis.

1. Supporting Figures:

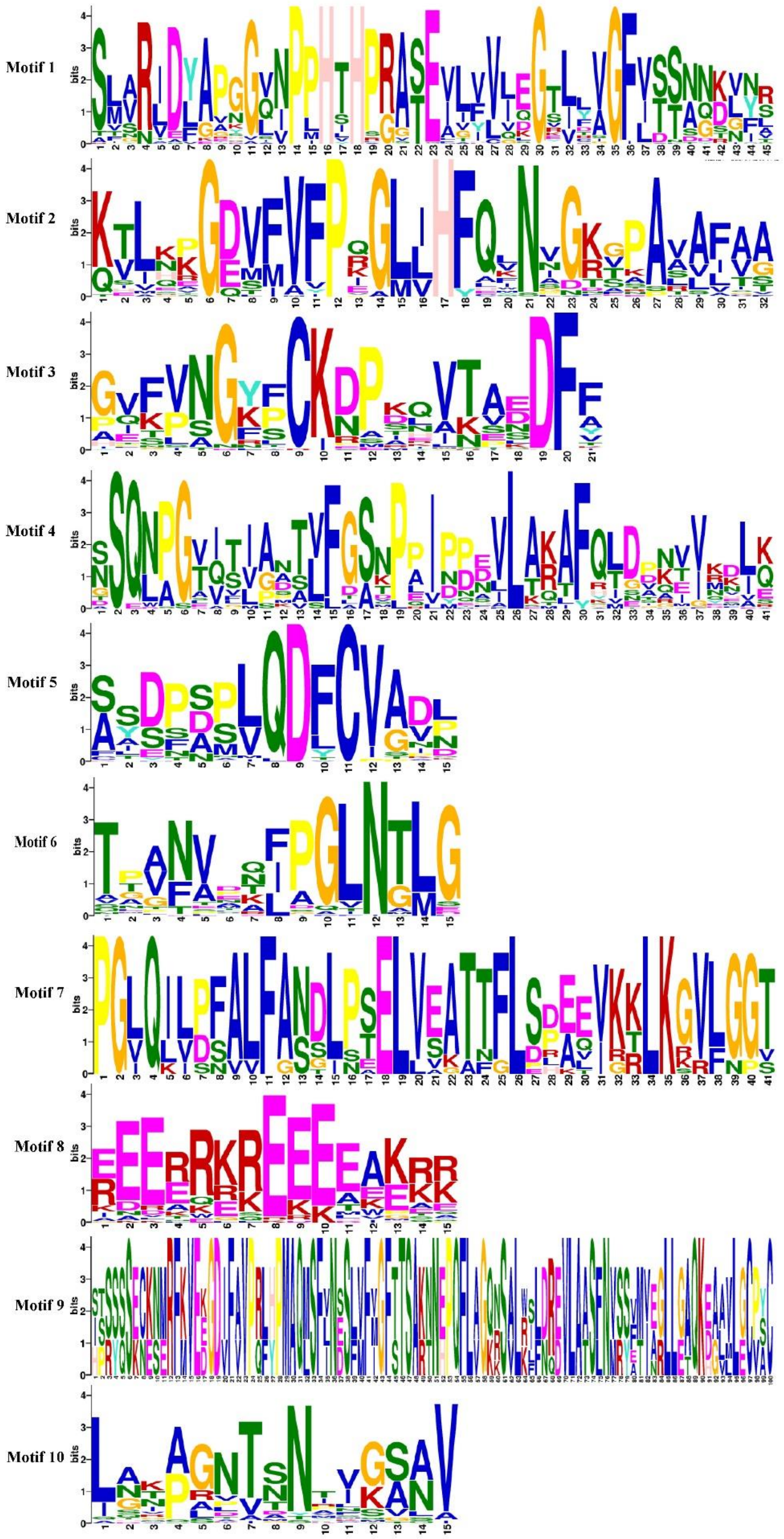

Figure S1. The 10 conserved motifs of rapeseed *BnGLPs*.

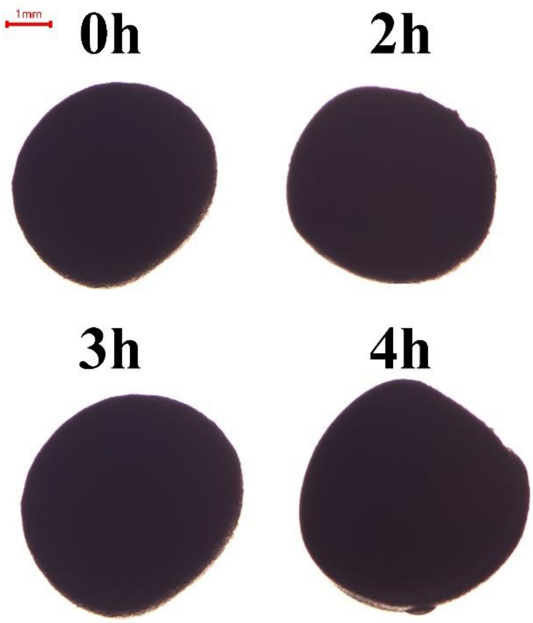

Figure S2. 0-4 h seed germination of ZS11. 0 h refer to dry seed, 2-4 h refer to seed imbibed in water with 2, 3, 4 hours, respectively.

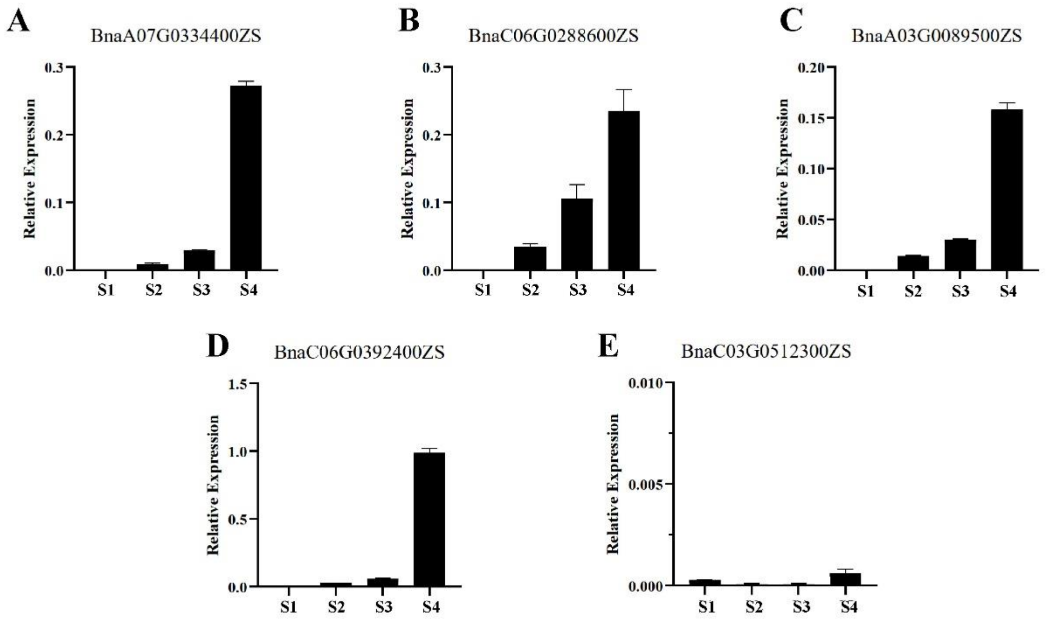

Figure S3. Relative expression levels of 5 *BnGLPs* in different state of axillary buds. S1 is state of dormant axillary buds, S2 is state of temporarily dormant axillary buds, S3 is state of being activated axillary buds, and S4 is state of elongating axillary buds. Error bars are standard deviations of three biological replicates.

2. **Supporting Tables:**

Table S1 Segmental duplication and tandem duplication gene pairs among *BnGLPs* family members.

No.	Segmental duplication gene pairs	Tandem duplication gene pairs
1	BnaA07G0174100ZS/BnaC06G0166900ZS	BnaA04G0132400ZS/BnaA04G0132500ZS
2	BnaA04G0105200ZS/BnaC04G0388100ZS	BnaC02G0102600ZS/BnaC02G0102700ZS
3	BnaA08G0089800ZS/BnaC08G0128300ZS	BnaC08G0128300ZS/BnaC08G0128400ZS
4	BnaA04G0105200ZS/BnaA04G0133100ZS	
5	BnaA04G0105200ZS/BnaC04G0422000ZS	
6	BnaA04G0132400ZS/BnaC04G0421100ZS	
7	BnaA04G0133100ZS/BnaC04G0422000ZS	
8	BnaA04G0132400ZS/BnaA07G0169200ZS	
9	BnaA01G0394400ZS/BnaC01G0484900ZS	
10	BnaA01G0394400ZS/BnaA05G0470500ZS	
11	BnaA05G0470500ZS/BnaC01G0484900ZS	
12	BnaC08G0194800ZS/BnaC08G0524900ZS	
13	BnaA06G0057800ZS/BnaC08G0524900ZS	
14	BnaC05G0073000ZS/BnaC08G0524900ZS	
15	BnaA07G0169200ZS/BnaC06G0159700ZS	
16	BnaA06G0057800ZS/BnaC08G0194800ZS	
17	BnaC05G0073000ZS/BnaC08G0194800ZS	
18	BnaA10G0010600ZS/BnaC05G0012500ZS	
19	BnaA06G0057800ZS/BnaC05G0073000ZS	
20	BnaA09G0561300ZS/BnaC08G0410100ZS	
21	BnaA05G0470800ZS/BnaC01G0485000ZS	
22	BnaA05G0470800ZS/BnaA06G0343700ZS	
23	BnaA05G0470800ZS/BnaC03G0360600ZS	
24	BnaC01G0485000ZS/BnaC03G0360600ZS	
25	BnaA03G0089500ZS/BnaC03G0101700ZS	
26	BnaA03G0089500ZS/BnaC09G0459300ZS	
27	BnaA03G0089500ZS/BnaC02G0102600ZS	
28	BnaA02G0086600ZS/BnaA03G0089500ZS	
29	BnaA07G0258500ZS/BnaC06G0392400ZS	
30	BnaA07G0334400ZS/BnaC06G0392400ZS	
31	BnaC06G0288600ZS/BnaC06G0392400ZS	
32	BnaA02G0201400ZS/BnaC06G0392400ZS	
33	BnaC02G0268300ZS/BnaC06G0392400ZS	
34	BnaA07G0258500ZS/BnaA07G0334400ZS	
35	BnaA07G0258500ZS/BnaC06G0288600ZS	
36	BnaA02G0201400ZS/BnaA07G0258500ZS	
37	BnaA07G0258500ZS/BnaC02G0268300ZS	

38	BnaA08G0184000ZS/BnaC07G0533100ZS
39	BnaA08G0184000ZS/BnaC03G0692500ZS
40	BnaA03G0556700ZS/BnaA08G0184000ZS
41	BnaC03G0692500ZS/BnaC07G0533100ZS
42	BnaC03G0512300ZS/BnaC07G0533100ZS
43	BnaA03G0556700ZS/BnaC07G0533100ZS
44	BnaA07G0334400ZS/BnaC06G0288600ZS
45	BnaA07G0334400ZS/BnaC02G0268300ZS
46	BnaA02G0201400ZS/BnaC06G0288600ZS
47	BnaC02G0268300ZS/BnaC06G0288600ZS
48	BnaA03G0556700ZS/BnaC03G0692500ZS
49	BnaA10G0172500ZS/BnaC03G0101700ZS
50	BnaC03G0101700ZS/BnaC09G0459300ZS
51	BnaC02G0102600ZS/BnaC03G0101700ZS
52	BnaA02G0086600ZS/BnaC03G0101700ZS
53	BnaA10G0172500ZS/BnaC09G0459300ZS
54	BnaA10G0172500ZS/BnaC02G0102600ZS
55	BnaA02G0086600ZS/BnaA10G0172500ZS
56	BnaC02G0102600ZS/BnaC09G0459300ZS
57	BnaA02G0086600ZS/BnaC09G0459300ZS
58	BnaA02G0086600ZS/BnaC02G0102600ZS
59	BnaA06G0311200ZS/BnaC03G0512300ZS
60	BnaA03G0556700ZS/BnaA06G0311200ZS
61	BnaA01G0381600ZS/BnaC01G0479300ZS
62	BnaA02G0201400ZS/BnaC02G0268300ZS
No.	BnaA09G0611200ZS/BnaC08G0465400ZS
63	BnaA09G0611200ZS/BnaC05G0159400ZS
64	BnaA06G0132400ZS/BnaA09G0611200ZS
65	BnaC05G0159400ZS/BnaC08G0465400ZS
66	BnaA06G0132400ZS/BnaC08G0465400ZS
67	BnaA06G0132400ZS/BnaC05G0159400ZS
68	BnaA07G0249800ZS/BnaC05G0159400ZS
69	BnaC05G0159400ZS/BnaC06G0275500ZS
70	BnaA06G0132400ZS/BnaA07G0249800ZS
71	BnaA06G0132400ZS/BnaC06G0275500ZS
72	BnaA09G0657900ZS/BnaC08G0519500ZS
73	BnaA03G0412900ZS/BnaC07G0384800ZS
74	BnaA02G0286100ZS/BnaA06G0437800ZS
75	BnaA02G0286100ZS/BnaC05G0023700ZS
76	BnaA02G0286100ZS/BnaC02G0388400ZS
77	BnaA06G0437800ZS/BnaC05G0023700ZS
78	BnaA06G0437800ZS/BnaC02G0388400ZS
79	BnaA07G0249800ZS/BnaC06G0275500ZS
80	BnaC02G0388400ZS/BnaC05G0023700ZS

Table S2. Ka/Ks analysis for paralogous gene pairs of *BnGLPs* in *B. napus*.

Gene 1	Gene 2	Ka	Ks	Ka_Ks
BnaA04G0132400ZS	BnaC04G0421100ZS	0.0441	0.0535	0.8255
BnaC04G0421100ZS	BnaA04G0132400ZS	0.0441	0.0535	0.8255
BnaA09G0184900ZS	BnaC09G0210700ZS	0.0080	0.0123	0.6480
BnaC09G0210700ZS	BnaA09G0184900ZS	0.0080	0.0123	0.6480
BnaA02G0086700ZS	BnaA02G0086600ZS	0.1158	0.2451	0.4726
BnaA04G0132500ZS	BnaC04G0421100ZS	0.0311	0.0692	0.4497
BnaC04G0421100ZS	BnaA04G0132500ZS	0.0311	0.0692	0.4497
BnaA02G0201400ZS	BnaC02G0268300ZS	0.0291	0.0678	0.4288
BnaC02G0268300ZS	BnaA02G0201400ZS	0.0291	0.0678	0.4288
BnaA07G0249800ZS	BnaC06G0275500ZS	0.0426	0.1009	0.4222
BnaC06G0275500ZS	BnaA07G0249800ZS	0.0426	0.1009	0.4222
BnaA05G0470800ZS	BnaC03G0360600ZS	0.0870	0.2085	0.4171
BnaC03G0360600ZS	BnaA05G0470800ZS	0.0870	0.2085	0.4171
BnaC02G0102700ZS	BnaA02G0086600ZS	0.1184	0.3081	0.3844
BnaA05G0470800ZS	BnaC01G0485000ZS	0.1055	0.2761	0.3819
BnaC01G0485000ZS	BnaA05G0470800ZS	0.1055	0.2761	0.3819
BnaC02G0103000ZS	BnaA02G0086600ZS	0.1245	0.3428	0.3633
BnaA09G0561300ZS	BnaC08G0410100ZS	0.0681	0.1903	0.3577
BnaC08G0410100ZS	BnaA09G0561300ZS	0.0681	0.1903	0.3577
BnaA02G0086900ZS	BnaA02G0086600ZS	0.1245	0.3529	0.3529
BnaC05G0513100ZS	BnaC08G0519500ZS	0.0280	0.0795	0.3520
BnaC08G0519500ZS	BnaC05G0513100ZS	0.0280	0.0795	0.3520
BnaA07G0169200ZS	BnaC09G0210700ZS	0.1750	0.5255	0.3329
BnaA07G0169200ZS	BnaA09G0184900ZS	0.1708	0.5331	0.3205
BnaA07G0169200ZS	BnaC06G0159700ZS	0.0142	0.0444	0.3199
BnaC06G0159700ZS	BnaA07G0169200ZS	0.0142	0.0444	0.3199
BnaC02G0102700ZS	BnaC02G0103000ZS	0.0853	0.2837	0.3008
BnaC02G0103000ZS	BnaC02G0102700ZS	0.0853	0.2837	0.3008
BnaA02G0086900ZS	BnaC02G0102700ZS	0.0853	0.2930	0.2913
BnaC02G0102700ZS	BnaA02G0086900ZS	0.0853	0.2930	0.2913
BnaC01G0485000ZS	BnaC03G0360600ZS	0.0810	0.2870	0.2824
BnaC03G0360600ZS	BnaC01G0485000ZS	0.0810	0.2870	0.2824
BnaA02G0086700ZS	BnaC02G0103000ZS	0.0829	0.3035	0.2732
BnaC02G0103000ZS	BnaA02G0086700ZS	0.0829	0.3035	0.2732
BnaA02G0086700ZS	BnaA02G0086900ZS	0.0829	0.3130	0.2649
BnaA02G0086900ZS	BnaA02G0086700ZS	0.0829	0.3130	0.2649
BnaC08G0128300ZS	BnaC09G0210700ZS	0.1242	0.5886	0.2109
BnaA07G0334400ZS	BnaC06G0392400ZS	0.0266	0.1284	0.2068
BnaC06G0392400ZS	BnaA07G0334400ZS	0.0266	0.1284	0.2068
BnaA08G0089800ZS	BnaC09G0210700ZS	0.1283	0.6207	0.2066
BnaA06G0057800ZS	BnaC05G0073000ZS	0.0232	0.1137	0.2042
BnaC05G0073000ZS	BnaA06G0057800ZS	0.0232	0.1137	0.2042

BnaC08G0128300ZS	BnaA09G0184900ZS	0.1171	0.5876	0.1993
BnaA09G0657900ZS	BnaC08G0519500ZS	0.0248	0.1246	0.1992
BnaC08G0519500ZS	BnaA09G0657900ZS	0.0248	0.1246	0.1992
BnaA06G0057800ZS	BnaC08G0194800ZS	0.0576	0.2937	0.1962
BnaC08G0194800ZS	BnaA06G0057800ZS	0.0576	0.2937	0.1962
BnaA08G0089800ZS	BnaA09G0184900ZS	0.1212	0.6197	0.1956
BnaA09G0184900ZS	BnaC04G0421100ZS	0.1256	0.6841	0.1835
BnaC04G0421100ZS	BnaC09G0210700ZS	0.1243	0.6777	0.1835
BnaC09G0210700ZS	BnaC04G0421100ZS	0.1243	0.6777	0.1835
BnaA06G0132400ZS	BnaC05G0159400ZS	0.0200	0.1101	0.1819
BnaC05G0159400ZS	BnaA06G0132400ZS	0.0200	0.1101	0.1819
BnaC05G0073000ZS	BnaC08G0194800ZS	0.0521	0.2998	0.1738
BnaC08G0194800ZS	BnaC05G0073000ZS	0.0521	0.2998	0.1738
BnaC08G0194800ZS	BnaC08G0524900ZS	0.0524	0.3190	0.1642
BnaC08G0524900ZS	BnaC08G0194800ZS	0.0524	0.3190	0.1642
BnaA02G0201400ZS	BnaA07G0258500ZS	0.0585	0.3602	0.1625
BnaA07G0258500ZS	BnaA02G0201400ZS	0.0585	0.3602	0.1625
BnaA06G0132400ZS	BnaA09G0611200ZS	0.0607	0.3880	0.1564
BnaA09G0611200ZS	BnaA06G0132400ZS	0.0607	0.3880	0.1564
BnaA06G0057800ZS	BnaC08G0524900ZS	0.0507	0.3291	0.1542
BnaC08G0524900ZS	BnaA06G0057800ZS	0.0507	0.3291	0.1542
BnaA06G0132400ZS	BnaC08G0465400ZS	0.0619	0.4016	0.1542
BnaC08G0465400ZS	BnaA06G0132400ZS	0.0619	0.4016	0.1542
BnaA07G0258500ZS	BnaC06G0288600ZS	0.0110	0.0712	0.1542
BnaC06G0288600ZS	BnaA07G0258500ZS	0.0110	0.0712	0.1542
BnaA10G0010600ZS	BnaC05G0012500ZS	0.0102	0.0661	0.1536
BnaC05G0012500ZS	BnaA10G0010600ZS	0.0102	0.0661	0.1536
BnaA07G0258500ZS	BnaC02G0268300ZS	0.0523	0.3442	0.1519
BnaC02G0268300ZS	BnaA07G0258500ZS	0.0523	0.3442	0.1519
BnaA02G0201400ZS	BnaC06G0288600ZS	0.0510	0.3380	0.1509
BnaC06G0288600ZS	BnaA02G0201400ZS	0.0510	0.3380	0.1509
BnaA02G0375300ZS	BnaC04G0422000ZS	0.0079	0.0524	0.1503
BnaC04G0422000ZS	BnaA02G0375300ZS	0.0079	0.0524	0.1503
BnaA07G0174100ZS	BnaC06G0166900ZS	0.0100	0.0678	0.1478
BnaC06G0166900ZS	BnaA07G0174100ZS	0.0100	0.0678	0.1478
BnaC02G0268300ZS	BnaC06G0288600ZS	0.0453	0.3241	0.1396
BnaC06G0288600ZS	BnaC02G0268300ZS	0.0453	0.3241	0.1396
BnaA04G0132400ZS	BnaC09G0210700ZS	0.0877	0.6350	0.1381
BnaC09G0210700ZS	BnaA04G0132400ZS	0.0877	0.6350	0.1381
BnaA04G0133100ZS	BnaC06G0166900ZS	0.1048	0.7656	0.1368
BnaC06G0166900ZS	BnaA04G0133100ZS	0.1048	0.7656	0.1368
BnaC04G0422000ZS	BnaC06G0166900ZS	0.1094	0.8024	0.1363
BnaA09G0184900ZS	BnaA04G0132400ZS	0.0877	0.6482	0.1353
BnaC04G0388100ZS	BnaA04G0133100ZS	0.1132	0.8427	0.1343

BnaA02G0375300ZS	BnaC06G0166900ZS	0.1058	0.7961	0.1330
BnaA02G0201400ZS	BnaA07G0334400ZS	0.0858	0.6567	0.1306
BnaA04G0132500ZS	BnaC09G0210700ZS	0.0852	0.6630	0.1286
BnaC09G0210700ZS	BnaA04G0132500ZS	0.0852	0.6630	0.1286
BnaA08G0089800ZS	BnaC08G0128300ZS	0.0040	0.0317	0.1271
BnaC08G0128300ZS	BnaA08G0089800ZS	0.0040	0.0317	0.1271
BnaC04G0422000ZS	BnaA07G0174100ZS	0.1047	0.8248	0.1269
BnaA09G0184900ZS	BnaA04G0132500ZS	0.0853	0.6768	0.1260
BnaA04G0133100ZS	BnaA07G0174100ZS	0.1001	0.8055	0.1243
BnaA07G0174100ZS	BnaA04G0133100ZS	0.1001	0.8055	0.1243
BnaA09G0657900ZS	BnaC05G0513100ZS	0.0144	0.1169	0.1232
BnaC05G0513100ZS	BnaA09G0657900ZS	0.0144	0.1169	0.1232
BnaA04G0133100ZS	BnaC04G0422000ZS	0.0039	0.0322	0.1219
BnaC04G0422000ZS	BnaA04G0133100ZS	0.0039	0.0322	0.1219
BnaA02G0375300ZS	BnaA04G0133100ZS	0.0039	0.0323	0.1215
BnaA04G0133100ZS	BnaA02G0375300ZS	0.0039	0.0323	0.1215
BnaC03G0101700ZS	BnaC09G0459300ZS	0.0356	0.2945	0.1208
BnaC09G0459300ZS	BnaC03G0101700ZS	0.0356	0.2945	0.1208
BnaA02G0375300ZS	BnaA07G0174100ZS	0.1012	0.8379	0.1208
BnaA09G0611200ZS	BnaC05G0159400ZS	0.0472	0.3996	0.1182
BnaC05G0159400ZS	BnaA09G0611200ZS	0.0472	0.3996	0.1182
BnaA03G0412900ZS	BnaC07G0384800ZS	0.0086	0.0729	0.1179
BnaC07G0384800ZS	BnaA03G0412900ZS	0.0086	0.0729	0.1179
BnaC05G0159400ZS	BnaC08G0465400ZS	0.0495	0.4517	0.1096
BnaC08G0465400ZS	BnaC05G0159400ZS	0.0495	0.4517	0.1096
BnaA04G0105200ZS	BnaA04G0133100ZS	0.1116	1.0192	0.1095
BnaA04G0132400ZS	BnaA04G0132500ZS	0.0041	0.0373	0.1087
BnaA04G0132500ZS	BnaA04G0132400ZS	0.0041	0.0373	0.1087
BnaA07G0174100ZS	BnaC04G0388100ZS	0.0696	0.6643	0.1048
BnaC04G0388100ZS	BnaA07G0174100ZS	0.0696	0.6643	0.1048
BnaC05G0073000ZS	BnaC08G0524900ZS	0.0433	0.4179	0.1035
BnaC08G0524900ZS	BnaC05G0073000ZS	0.0433	0.4179	0.1035
BnaC04G0388100ZS	BnaC06G0166900ZS	0.0675	0.6620	0.1020
BnaC06G0166900ZS	BnaC04G0388100ZS	0.0675	0.6620	0.1020
BnaA03G0089500ZS	BnaC09G0459300ZS	0.0322	0.3202	0.1006
BnaC09G0459300ZS	BnaA03G0089500ZS	0.0322	0.3202	0.1006
BnaA10G0172500ZS	BnaC03G0101700ZS	0.0367	0.3682	0.0996
BnaC03G0101700ZS	BnaA10G0172500ZS	0.0367	0.3682	0.0996
BnaC06G0392400ZS	BnaA07G0258500ZS	0.0535	0.5448	0.0981
BnaA07G0334400ZS	BnaC02G0268300ZS	0.0642	0.6566	0.0978
BnaC02G0268300ZS	BnaA07G0334400ZS	0.0642	0.6566	0.0978
BnaC06G0392400ZS	BnaC02G0268300ZS	0.0682	0.7007	0.0973
BnaA04G0105200ZS	BnaA07G0174100ZS	0.0685	0.7088	0.0966
BnaA07G0174100ZS	BnaA04G0105200ZS	0.0685	0.7088	0.0966

BnaA04G0105200ZS	BnaC06G0166900ZS	0.0663	0.7062	0.0939
BnaC06G0166900ZS	BnaA04G0105200ZS	0.0663	0.7062	0.0939
BnaA07G0258500ZS	BnaA07G0334400ZS	0.0484	0.5542	0.0873
BnaA07G0334400ZS	BnaA07G0258500ZS	0.0484	0.5542	0.0873
BnaA03G0089500ZS	BnaA10G0172500ZS	0.0333	0.3863	0.0862
BnaA10G0172500ZS	BnaA03G0089500ZS	0.0333	0.3863	0.0862
BnaC06G0288600ZS	BnaC06G0392400ZS	0.0465	0.5569	0.0835
BnaC06G0392400ZS	BnaC06G0288600ZS	0.0465	0.5569	0.0835
BnaA04G0105200ZS	BnaC04G0388100ZS	0.0039	0.0469	0.0834
BnaC04G0388100ZS	BnaA04G0105200ZS	0.0039	0.0469	0.0834
BnaA07G0334400ZS	BnaC06G0288600ZS	0.0461	0.5663	0.0814
BnaA03G0089500ZS	BnaC03G0101700ZS	0.0159	0.1991	0.0800
BnaC03G0101700ZS	BnaA03G0089500ZS	0.0159	0.1991	0.0800
BnaA02G0086600ZS	BnaC03G0101700ZS	0.0301	0.3794	0.0792
BnaC03G0101700ZS	BnaA02G0086600ZS	0.0301	0.3794	0.0792
BnaA02G0086700ZS	BnaC02G0102700ZS	0.0082	0.1042	0.0784
BnaC02G0102700ZS	BnaA02G0086700ZS	0.0082	0.1042	0.0784
BnaA02G0086600ZS	BnaC09G0459300ZS	0.0268	0.3636	0.0737
BnaC09G0459300ZS	BnaA02G0086600ZS	0.0268	0.3636	0.0737
BnaA02G0086600ZS	BnaA03G0089500ZS	0.0300	0.4254	0.0705
BnaA03G0089500ZS	BnaA02G0086600ZS	0.0300	0.4254	0.0705
BnaA01G0381600ZS	BnaC01G0479300ZS	0.0058	0.0852	0.0684
BnaC01G0479300ZS	BnaA01G0381600ZS	0.0058	0.0852	0.0684
BnaC02G0102600ZS	BnaC03G0101700ZS	0.0279	0.4215	0.0662
BnaC02G0102600ZS	BnaC09G0459300ZS	0.0246	0.3834	0.0642
BnaA09G0611200ZS	BnaC08G0465400ZS	0.0063	0.1054	0.0596
BnaC08G0465400ZS	BnaA09G0611200ZS	0.0063	0.1054	0.0596
BnaA01G0394400ZS	BnaC01G0484900ZS	0.0058	0.1078	0.0540
BnaC01G0484900ZS	BnaA01G0394400ZS	0.0058	0.1078	0.0540
BnaA10G0172500ZS	BnaC02G0102600ZS	0.0257	0.4908	0.0524
BnaC02G0102600ZS	BnaA10G0172500ZS	0.0257	0.4908	0.0524
BnaA02G0086600ZS	BnaC02G0102600ZS	0.0021	0.0592	0.0356
BnaC02G0102600ZS	BnaA02G0086600ZS	0.0021	0.0592	0.0356
BnaA02G0086900ZS	BnaC02G0103000ZS	0	0.0063	0
BnaA10G0172500ZS	BnaC09G0459300ZS	0	0.1309	0
BnaC02G0103000ZS	BnaA02G0086900ZS	0	0.0063	0
BnaC09G0459300ZS	BnaA10G0172500ZS	0	0.1309	0

Table S3. ZS11 *BnGLPs* gene-specific primers used for RT-qPCR analysis.

Gene Name	Forward Primer (5′→3′) Sequence	Reverse Primer (5′→3′) Sequence
Bnaactin7	CCCTGGAATTGCTGACCGTA	TGGAAAGTGCTGAGGGATGC
BnaA07G0334400ZS	CGCAGGGCTTGCTTCATTTC	TTGACTGTAGCAGGCGGTAG
BnaC06G0288600ZS	CTTAGGCACTCCTGGAAACAC	CCCGCATTGATCTGGAAG
BnaA02G0286100ZS	GCTACGCTGCTCTGGTGTCT	TGGTGATGAGTCCTGGAAAGT
BnaA03G0089500ZS	GTCATTCCTCTTCACACTCACTC	CCCTTCCCAGAGTTGAGC
BnaC06G0392400ZS	CAGATCAATGCGGGTAAGAGC	GAAAGTAGTAGCCGAGACGAGC
BnaC03G0512300ZS	CTCAGTCGCCACTTCTGGTC	TTCAAAACCCCAGTACCAGTGT
